# Reaction-diffusion modeling in the NEURON simulator

**DOI:** 10.1186/1471-2202-13-S1-P119

**Published:** 2012-07-16

**Authors:** Robert A McDougal, Yosef Skolnick, James C Schaff, William W Lytton, Michael L Hines

**Affiliations:** 1 Department of Neurobiology, Yale University, New Haven, CT 06520, USA; 2 Department of Physiology & Pharmacology, SUNY Downstate Medical Center, Brooklyn, NY 11203, USA; 3 Department of Computer Science, CUNY Brooklyn College, Brooklyn, NY 11210, USA; 4 Center for Cell Analysis & Modeling, University of Connecticut Health Center, Farmington, CT 06030, USA; 5 Kings County Hospital, Brooklyn, NY 11203, USA

## 

The NEURON simulator is a widely used tool for studying detailed single cell and network models. In recognition of the growing importance of multi-scale modeling, we have expanded NEURON’s support for intracellular chemical dynamics. In particular, we discuss our work with stochastic reaction-diffusion models and three-dimensional simulations.

Unlike previous NEURON mechanisms, arbitrary new reaction schemes may be specified at run-time via HOC or Python; no separate compilation step is required. This flexibility allows us to import models written in the Systems Biology Markup Language (SBML), which will facilitate collaboration between the neuroscience and cell biology communities.

In certain situations, such as calcium dynamics near a spine, only a few particles of a given chemical species are present. As these particles randomly move around, there is the potential for large percentage deviations from the mean concentration. To study these effects, we support the Gillespie and tau-leaping algorithms for stochastic reaction-diffusion, and our methods can easily be extended to other compartment-based approaches. Deterministic diffusion is also supported.

For electrophysiological simulations, NEURON employs a one-dimensional approximation of neuronal morphologies, but certain chemical reaction-diffusion problems in neurons exhibit behavior that cannot be captured by a such an approximation due to a small space constant. Many previous modelers have implemented radial diffusion as well as longitudinal diffusion, but this too misses many biologically important phenomena such as the nature of calcium microdomains near a spine or diffusion into the soma. Spatial simulations present additional challenges not present in one-dimensional approximations: geometric details can no longer be ignored; for example, a spatial simulator must define the shape of the joins between dendritic sections. Constructing a mesh becomes more complicated. For performance reasons, we allow different species to use different meshes, but this requires care when transferring data between grids. We discuss these problems and present our current strategies for resolving them.

We show preliminary modeling results illustrating the utility of these techniques to study the interaction of chemical and electrical dynamics in neuronal information processing.

